# Supplementary material for: The Role of MaFAD2 Gene in Bud Dormancy and Cold Resistance in Mulberry Trees (Morus alba L.)
Source: Int J Mol Sci. 2024 Dec 12;25(24):13341. doi: 10.3390/ijms252413341 (PMC11728178; doi:10.3390/ijms252413341)
Supplement: Supplementary file 1 [file ijms-25-13341-s001.zip › 20241204 zhao et al. Supplementary file S1.pdf]

MaFAD2

>NS14_07T001899.1   CDS=1-1659

ATGGTGAAAGGGCTTGCTTGGGTTGTGTGTGTTTATGGAGTGCCATTGTTGGTGGTGAA
TGGCTTTCTGGTGTTGATCACTTTTTTGCACCACACTCACCCTTCGTTGCCTCATTACGA
TTCGTCCGAATGGGACTGGTTGAGAGGAGCCTTGGCCACCGTGGATAGAGACTATAGG
CTGTTGAACAAGGTTTTCCATAACATCACGGACACTCATGTAGCTCACCATTTGTTCTC
AACTATGCCGCATTATCATGCCATGGAGGCCACCAAGGCGATCAAGCCGATACTCGGAG
ATTACTATCAGTTCGATGGGACGCCAGTGTACAAAGCAATGTTTAGGGAGACCAAGGA
GTGTGTCTTTGTTGAGCCCGATGAAAGTGATGAGAAAGGCGTCTTCTGGTACCAGAAG
TTTGTTTCTGTTGCTTGGAAACCTCCACCTAGAGAGAAACTAGAGAGAGAGAAGCTAG
AGAGAGAAAGTCCCAGAGCTTCTTCAATCTCAGGTTTTGGAACAATGGGTGCCGATGG
CCGAATGTCAGTTCCCCCAGCCGCGAAAAACTCAGAAATTGACAGCCTCAAGCGAGT
CCCCTACTCAAAGCCCCCATTCACACTTAGCCAAATCAAGAAAGCCATCCCACCCCATT
GCTTTAAACGCTCTGTCGTACACTCATTCTCTTATGTCGTTTACGACCTCACCATTGCCT
TCATCTTCTACTACATCGTCACCAATTACATTCCCCAATTGCCTCACCCTCTCCCTTACCT
GGCCTGGCCCATTTACGGCTTCATCCAAGGCTGTGTCCTCACCGGTGTTTGGGTCATAG
CCCACGAATGTGGGCACCACGCCTTTAGTGACTACCAATGGCTTGACGACACCGTGGG
CCTAATCCTCCACTCTTGTCTTCTAGTCCCTTACTTTTCATGGAAATACAGCCACCGCCA
TCACCATTCAAACACAGGCTCTCTTGAGCGTGATGAAGTCTTTGTCCCCAAGCGAAAG
TCTAGCATAAGATGGTACTCCAAATATCTCAACAACCCACTTGGCAGATTTCTCACCCTT
ACTATCACACTCACTCTAGGATGGCCTTTGTACCTGCTGTTCAACGTTTCAGGCAAACC
TTATGATCGTTTTGCATGCCACTTTGACCCTTATGGCCCCATCTACTCGGACCGTGAGAG
GCTACAGATTTACATCTCAGACACGGGCATTCTCGCTGTCTGCTACGGACTTTACCGCC
TCACCATGGTGAAAGGGCTTGCTTGGGTTGTGTGTGTTTATGGAGTGCCATTGTTGGTG
GTAAATGGCTTTCTGGTGTTGATCACTTTCTTGCAGCATACTCACCCTTCGTTGCCTCAT
TACGATTCGTCCGAATGGGACTGGTTGAGAGGAGCCTTGGCCACCGTGGACAGAGACT
ATGGGCTGTTGAACAAGGTTTTCCATAACATCACGGACACTCATGTAGCTCACCATTTG
TTCTCAACTATGCCGCATTATCATGCCATGGAGGCCACCAAGGCGATTAAGCCGATACT
CGGAGATTACTATCAGTTCGATGGGACGCCGGTGTACAATGCAATGTTTAGGGAGACC
AAGGAGTGTGTCTTTGTTGAGCCCGATGAAAGTGATGAGAAAGGCGTCTTCTGGTACA
AGAAGTTATGA
